# Studies of Secondary Melanoma on C57BL/6J Mouse Liver Using ^1^H NMR Metabolomics

**DOI:** 10.3390/metabo3041011

**Published:** 2013-10-31

**Authors:** Ju Feng, Nancy G. Isern, Sarah D. Burton, Jian Zhi Hu

**Affiliations:** Pacific Northwest National Laboratory, 902 Battelle Blvd, Richland, WA 99352, USA; E-Mails: ju.feng@pnnl.gov (J.F.); nancy.isern@pnnl.gov (N.I.); sarah.burton@pnnl.gov (S.D.B.)

**Keywords:** ^1^H HR-MAS, NMR, B16-F10 melanoma, metabolomics, multivariate analysis, PCA

## Abstract

NMR metabolomics, consisting of solid state high resolution magic angle spinning (HR-MAS) ^1^H-NMR, liquid state high resolution ^1^H-NMR, and principal components analysis (PCA) has been used to study secondary metastatic B16-F10 melanoma in C57BL/6J mouse liver. The melanoma group can be differentiated from its control group by PCA analysis of the estimates of absolute concentrations from liquid state ^1^H-NMR spectra on liver tissue extracts or by the estimates of absolute peak intensities of metabolites from ^1^H HR-MAS-NMR data on intact liver tissues. In particular, we found that the estimates of absolute concentrations of glutamate, creatine, fumarate and cholesterol are elevated in the melanoma group as compared to controls, while the estimates of absolute concentrations of succinate, glycine, glucose, and the family of linear lipids including long chain fatty acids, total choline and acyl glycerol are decreased. The ratio of glycerophosphocholine (GPC) to phosphocholine (PCho) is increased by about 1.5 fold in the melanoma group, while the estimate of absolute concentration of total choline is actually lower in melanoma mice. These results suggest the following picture in secondary melanoma metastasis: Linear lipid levels are decreased by beta oxidation in the melanoma group, which contributes to an increase in the synthesis of cholesterol, and also provides an energy source input for TCA cycle. These findings suggest a link between lipid oxidation, the TCA cycle and the hypoxia-inducible factors (HIF) signal pathway in tumor metastases. Thus, this study indicates that the metabolic profile derived from NMR analysis can provide a valuable bio-signature of malignancy and cell hypoxia in metastatic melanoma.

## 1. Introduction

Metabolic changes are among the earliest cellular responses to environmental or physiological changes, and hence, metabolomics, studying the profile of metabolites, is a powerful systems biology tool that is capable of diagnosing a disease and evaluating the efficacy of a therapy in an early stage of illness. Nuclear Magnetic Resonance (NMR) spectroscopy is one of the leading metabolomics tools. Metabolomics analysis in biological and medicinal studies employing NMR in conjunction with statistical methods, especially multivariate data analysis, have proven to be a formidable tool to elucidate the complicated interactions in biological systems [1,2].

Melanoma is a malignant tumor of melanocytes [3], melanoma itself is treatable by surgical removal at early stage, *i.e.*, in cases without metastases, but is lethal when the cancer has spread into other organs and causes the majority (75%) of deaths related to skin cancer [4]. NMR based metabolomics have been extensively employed for monitoring metabolic changes in primary melanoma *in vivo* and *in vitro* [5,6,7,8,9,10,11]. However, little effort has been devoted to metabolic profiling of metastatic tumors in organs other than lymph nodes [10,11,12,13]. The mechanisms of metastases and proliferation are not fully understood, even though there is evidence that melanoma development is associated with HIF-1 (hypoxia-inducible factor 1) [14,15,16]. Animal models of melanoma have been instrumental in gaining the current level of understanding of the initiation, progression, and metastasis of melanoma. Although several animals models have been described, including guinea pig, opossum, and *Xiphophorus* fish, mouse models have provided the most significant and most recent advancements in metastatic melanoma research [17,18,19,20]. Melanoma cells firstly invaded the bloodstream or lymphatic vessels, then colonized the lung and further migrated to other far sites [21,22,23]. After 3–4 weeks of inoculation of B16 cells at C57BL/6J mice, histological studies revealed the presence of malignant melanoma and metastasis in liver, lungs and spleen, showing that the B16 mouse melanoma model is an easy to reproduce *in vivo* model of carcinogenesis [24,25], with liver presenting a moderate stasis and a spot-type tumor proliferation similar with lung tumor proliferation. There is no way to compensate for the absence of liver function in the long term when invaded by metastatic melanoma cells, since the liver has major roles in metabolism, such as detoxification, decomposition of red blood cells, glycogen storage, plasma protein synthesis, and hormone production.

In this study, we were interested in evaluating the metabolic changes in liver after the metastasis of primary melanoma cells implanted at the flank location on the legs of a mouse, *i.e.*, the metabolic effects of the secondary melanoma in liver confirmed by histological image under microscope. Both ^1^H HR-MAS NMR and liquid state ^1^H-NMR combined with multivariate statistics (principal components analysis, PCA) have been used for metabolic investigations in the livers excised from mice of both control and melanoma groups. The ultimate goal of the present study is to provide molecular information to identify metabolic pathways that are affected by melanoma metastasis in the liver. Hence, understanding the metabolic changes of metastatic melanoma would contribute both to a deeper knowledge of the underlying molecular processes in the pathological state and to the development of improved therapies. If successful, the results may provide new therapeutic targets for treating this fatal disease.

## 2. Materials and Methods

### 2.1. Animal Handling and Sample Preparation

Twelve 6 weeks old C57BL/6J male mice were purchased from Jackson Labs (SacramentoCA, USA) and housed at the Pacific Northwest National Laboratory animal facility. The mice were fed a standard diet and housed one mouse per cage. The animals were maintained in a temperature-controlled room (22–25 °C, 45% humidity) on a 12:12-h dark–light cycle and were allowed free access to water and food. The body weights of the mice were measured weekly. After acclimated for one week, the 12 animals were randomly separated into two groups with the control group containing five mice while the cancer group containing seven mice. At the age of seven weeks, the seven mice in the cancer group were subcutaneously injected with suspended 10^5^ B16-F10 tumor cells at the flank location on each leg. The five mice in the control group were injected with the same volume of PBS at the same place on each leg as the tumor group. At the age of 14 weeks, all the living animals (five mice from the cancer group and five from the control group) were sacrificed by CO_2_ asphyxiation and livers were excised and stored at −80 °C for NMR based metabolomics and morphology analyses. Note that among the seven mice in the cancer group, two were dead and were not used for the analysis. Specifically, for each mouse, the left liver lobe was cut in three parts. The upper left liver lobe was kept for ^1^H HR-MAS NMR analysis on intact tissues while the lower left liver lobe was kept for metabolite extraction for standard high resolution liquid state ^1^H-NMR metabolic profiling. The middle section of the left liver lobe was used for morphology analysis. All animal work was approved by the Institutional Animal Care and Use Committee (IACUC) at Pacific Northwest National Laboratory. Metastasized livers were checked under microscope to confirm the metastasis and the pathological images are given in the supplementary materials (Figure S1).

The modified Folch method was employed for tissue extraction by following established protocol [1,26], as this method generated the highest yields under mild extraction conditions [27,28]. It is commonly accepted that 95% or more of tissue lipids are extracted during the first step [29]. Generally, the metabolites were extracted by ice-cold MeOH-CHCl_3_-H_2_O using a mixture containing 250 µL methanol, 250 µL chloroform and water 175 µL for each 30–40 mg tissue. The extraction procedures are briefly described below. Step-1: about 30–40 mg liver tissue from the lower left liver lobe was homogenized inside a centrifugal plastic tube that was surrounded by an ice bath outside the tube to minimize metabolite changes after adding 4 mL MeOH and 0.85 mL H_2_O based on per gram of tissue, followed by vortexing the mixture and then 2 mL chloroform per gram of tissue was added, followed by vortexing again. Step-2: 2 mL chloroform and 2 mL H_2_O per gram of tissue were added in the mixture followed by vortexing, transferring the different layers into glass vials separately with syringes. Step-3: the solvents were removed by lyophilizer (MeOH/H_2_O layer, hydrophilic metabolites) or under nitrogen gas (CHCl_3_ layer, lipids metabolites) and the extracted metabolites were then stored at −80 °C until NMR measurements.

### 2.2. NMR Experiments

**^1^H HR-MAS:** Magic angle sample spinning (MAS) is a well recognized technique for obtaining high resolution ^1^H-NMR spectrum of metabolites of intact tissues without the need of extraction [5,6,7,8,9,10,11]. By spinning a sample tube containing biological tissues at a speed of a few kHz about an axis at an angle of 54.73 degrees relative to the main external magnetic field, line broadening due to magnetic susceptibility, chemical shift anisotropy interaction and residual homonuclear dipolar couplings are eliminated, resulting in a high resolution NMR spectrum directly on intact tissues. This method is termed as ^1^H HR-MAS [5,6,7,8,9,10,11]. The ^1^H HR-MAS NMR experiments on intact liver tissue of the upper left lobe excised from mice were performed on a Varian-Oxford Inova widebore 500 MHz (11.7 tesla) NMR spectrometer at 5 °C. A commercial 4.0 mm MAS probe from Varian was used. The sample spinning rate used was about 6 kHz. About 32–40 mg liver tissue was located between two home-made Kel-F plugs in the MAS rotor. The two sealed Kel-F plugs were inserted in the MAS rotor to prevent fluid leakage at a sample spinning rate of up to 8 kHz. A standard Varian PRESAT pulse sequence was used for the measurement with a single pulse excitation and 0.5 s low power pre-saturation at the H_2_O peak position for H_2_O suppression. The acquisition time was 1 s and the recycle delay time was 1 s, resulting in a total length of the pulse sequence of about 2.5 s. Each spectrum was acquired with a total of 1024 accumulations in about 50 min. We have found that under these experimental conditions, the ^1^H HR-MAS NMR spectrum of intact liver tissues was essentially unchanged (Figure S2), meaning biodegradation can be ignored during the 50 min data acquisition time. The spectrum was referenced to the β-glucose (4.64 ppm) in each spectrum in order to compare with the prior published results.

**High resolution liquid state ^1^H-NMR Experiments:** Shortly before the ^1^H-NMR metabolic profiling experiments, the water soluble metabolites were reconstituted in 600 µL of D_2_O containing 0.5 mM TSP. About 550 µL of the prepared sample was loaded into a standard 5 mm NMR tube (Wilmad, Vineland, NJ, USA) inside a cold room at 5 °C to minimize possible biodegradation in liquid state.

To further prevent biodegradation, 0.2% sodium azide (w/v) was added into the solution. Metabolic profiling experiments were carried out on a Varian 600 MHz NMR spectrometer equipped with a Z axis-gradient 5 mm HCN probe. All the NMR measurements were carried out at 20 °C. The standard Varian PRESAT pulse sequence using a single pulse excitation and 0.5 s low power pre-saturation at the H_2_O peak position for H_2_O suppression was used for the measurement. For acquiring each spectrum, an accumulation number of 1024 scans with acquisition time of 1 s and recycle delay time of 1.5 s were used, resulting in a total time of about 1 h for each sample.

Similar to the water soluble metabolites, shortly before the ^1^H-NMR metabolic profiling experiments, the lipid soluble metabolites were reconstituted in 600 µL of 600 µL CDCl_3_ (0.03% v/v TMS). About 550 µL of the prepared sample was loaded into a standard 5 mm NMR tube. A single pulse sequence was performed using 1024 scans at 20 °C. The acquisition time was 1 s and the recycle delay time was 1 s, resulting in a total experimental time of about 1 h. All other experimental procedures were identical to the water soluble metabolites discussed above.

**Table 1:** Metabolite Peak Assignments and Concentrations of Metabolites.

#	Peaks’ Signals	Chemical Shifts	Concentrations in NMR tube: (µM/mg) Mean ± SD		Estimates of absolute Concentrations in tissue: (mM) Mean ± SD	
		(ppm)				
			Tumor	Control	Tumor	Control
1	Cholesterol 18-CH3	0.67 (s)	1.03 ± 0.01	0.59 ± 0.09	0.62 ± 0.06	0.36 ± 0.06
2	Cholesterol 26, 27-CH3	0.87 (d)	1.03 ± 0.01	0.59 ± 0.09	0.62 ± 0.06	0.36 ± 0.06
3	Lipids CH3	0.88 (t)	1.43 ± 0.17	18.36 ± 0.72	8.56 ± 1.03	11.02 ± 0.43
4	Cholesterol 21-CH3	0.90 (d)	1.03 ± 0.10	0.59 ± 0.09	0.62 ± 0.06	0.36 ± 0.06
5	Ω3 CH3	0.97 (t)	4.36 ± 0.52	3.64 ± 0.26	2.62 ± 0.31	2.18 ± 0.16
6	Free cholesterol 19-CH3	1.01 (t)	1.03 ± 0.10	0.594 ± 0.092	0.62 ± 0.06	0.36 ± 0.06
7	Lipids CH2	1.27 (m)	58.66 ± 13.21	74.28 ± 2.38	35.19 ± 7.92	44.57 ± 1.43
8	Cholesterol CH2	1.45–1.50 (m)	1.03 ± 0.10	0.59 ± 0.09	0.62 ± 0.06	0.36 ± 0.06
9	Lipids CH2CH2CO	1.52–1.61 (m)	5.88 ± 1.26	7.99 ± 0.44	3.53 ± 0.75	4.79 ± 0.26
10	Lipids CH2CH=C	1.96–2.15 (m)	8.28 ± 1.51	11.33 ± 0.51	4.97 ± 0.90	6.80 ± 0.30
11	Lipids CH2CO	2.22–2.42 (m)	7.59 ± 1.67	9.62 ± 0.38	4.55 ± 1.00	5.77 ± 0.23
12	Lipids =CHCH2CH=	2.74–2.91 (m)	7.98 ± 1.74	8.71 ± 0.62	4.79 ± 1.04	5.23 ± 0.37
13	Choline N(CH3)3	3.36 (s)	28.81 ± 3.78	33.36 ± 1.06	17.29 ± 2.27	20.01 ± 0.64
14	Cholesterol 3-CHOH	3.53 (d)	0.71 ± 0.15	0.73 ± 0.02	0.43 ± 0.09	0.44 ± 0.01
15	Phosphatidylcholine N-CH2	3.81 (m)	3.44 ± 0.54	3.48 ± 0.26	2.06 ± 0.33	2.09 ± 0.16
16	Glycerophospholipid backbone 3-CH2	3.97 (m)	1.51 ± 0.44	2.05 ± 0.08	0.91 ± 0.26	1.23 ± 0.05
17	Glycerol backbone 1,3-CH2	4.16–4.30 (m)	0.15 ± 0.13	0.73 ± 0.09	0.09 ± 0.08	0.44 ± 0.05
18	Phosphatidylcholine PO-CH2	4.35 (m)	2.66 ± 0.44	2.70 ± 0.10	1.60 ± 0.27	1.62 ± 0.06
19	Esterified cholesterol 3-CHOH	4.72 (d)	0.55 ± 0.13	0.48 ± 0.06	0.33 ± 0.08	0.29 ± 0.04
20	Glycerophospholipid backbone 2-CH	5.22 (m)	1.51 ± 0.44	2.05 ± 0.08	0.91 ± 0.26	1.23 ± 0.05
21	Glycerol backbone 2-CH	5.28 (m)	0.037 ± 0.08	0.63 ± 0.37	0.02 ± 0.05	0.38 ± 0.22
22	Lipid CH=CH.	5.37 (m)	11.36 ± 2.57	13.18 ± 0.67	6.82 ± 1.54	7.91 ± 0.40
23	Leucine	0.99 (d), 1.70 (m), 3.72 (m)	1.17 ± 0.29	0.80 ± 0.27	0.70 ± 0.17	0.48 ± 0.16
24	Valine	0.97 (d), 1.02 (d), 2.28 (m), 3.61 (d)	0.84 ± 0.32	0.75 ± 0.39	0.50 ± 0.19	0.45 ± 0.23
25	Lactate	1.32 (d), 4.2 (q)	11.56 ± 5.00	18.58 ± 6.75	6.94 ± 3.00	11.15 ± 4.05
26	Alanine	1.47 (d), 3.77 (m)	6.59 ± 1.25	6.52 ± 2.23	3.95 ± 0.75	3.91 ± 1.338
27	Acetate	1.92 (s)	0.63 ± 0.20	0.27 ± 0.09	0.38 ± 0.12	0.16 ± 0.054
28	Glutamate	2.05 (m), 2.36 (dt), 3.76 (m)	3.64 ± 1.34	1.38 ± 0.33	2.18 ± 0.80	0.83 ± 0.20
29	Succinate	2.41 (s)	0.81 ± 0.11	1.11 ± 0.41	0.49 ± 0.07	0.67 ± 0.25
30	Glutamine	3.77 (m), 2.46 (m), 2.14 (m)	4.08 ± 1.40	6.34 ± 2.58	2.45 ± 0.84	3.80 ± 1.55
31	Glutathione	2.16 (m), 2.54 (m), 2.97 (m), 3.77 (m), 4.58 (dd)	2.64 ± 1.96	2.48 ± 1.42	1.58 ± 1.18	1.49 ± 0.85
32	Malate	2.35 (dd), 2.65 (dd), 4.26 (d)	2.17 ± 0.57	1.21 ± 0.53	1.30 ± 0.34	0.73 ± 0.32
33	Creatine + Creatinine	3.08 (s)	0.33 ± 0.12	0.32 ± 0.09	0.20 ± 0.072	0.19 ± 0.05
34	Choline	3.22–3.24, 3.50(m)	0.70 ± 0.10	0.65 ± 0.26	0.42 ± 0.06	0.39 ± 0.16
35	Taurine	3.25 (t), 3.40 (t)	21.95 ± 6.22	24.61 ± 6.23	13.17 ± 3.73	14.77 ± 3.74
36	Proline+Inositol	3.37 (m)	0.70 ± 0.42	0.90 ± 0.36	0.42 ± 0.25	0.54 ± 0.22
37	Glycine	3.55 (s)	2.68 ± 1.40	3.12 ± 1.78	1.61 ± 0.84	1.87 ± 1.07
38	â-Glucose	3.41 (dd), 3.47 (dd), 3.92 (m), 4.65 (d)	37.55 ± 25.83	74.75 ± 21.53	22.53 ± 15.50	44.85 ± 12.92
39	á-Glucose	3.42 (m), 3.55 (t), 3.76 (m) 3.81 (m), 3.92 (m), 5.23 (d)	0.80 ± 0.40	0.87 ± 0.44	0.48 ± 0.24	0.52 ± 0.26
40	Inosine Derivatives	4.42 (dd), 4.82 (t), 6.16 (d), 4.32 (m), 3.83 (dd), 3.91 (dd), 8.21 (s), 8.32 (s)				
41	Fumarate	6.75 (s)	0.23 ± 0.13	0.08 ± 0.02	0.14 ± 0.08	0.05 ± 0.012
42	ATP/ADP	4.22 (m), 4.37 (m), 4.57 (d), 6.13 (d), 8.26 (s), 8.52 (s)	1.12 ± 0.75	2.22 ± 0.56	0.67 ± 0.45	1.33 ± 0.34
43	Formate	8.34 (s)	0.63 ± 0.25	0.7 ± 0.28	0.38 ± 0.15	0.42 ± 0.17
44	-NH.	9.01				
45	sn-Glycero-3-phosphocholine	3.24, 3.50 (m)	1.25 ± 0.30	0.65 ± 0.16	0.75 ± 0.18	0.39 ± 0.10
46	2-Oxoglutarate	2.47 (t), 3.01 (t)	1.11 ± 0.42	0.85 ± 0.40	0.67 ± 0.25	0.51 ± 0.24
47	TMAO	3.25 (s)	1.02 ± 0.45	0.66 ± 0.25	0.61 ± 0.27	0.40 ± 0.15
48	O-Phosphocholine	3.23, 3.50 (m)	0.59 ± 0.34	0.44 ± 0.20	0.35 ± 0.20	0.26 ± 0.12
49	Hypoxanthine	8.15 (s)	0.70 ± 0.42	0.90 ± 0.36	0.42 ± 0.25	0.54 ± 0.22
50	Dimethylamine	2.71 (s)	0.12 ± 0.07	0.10 ± 0.01	0.07 ± 0.04	0.06 ± 0.01
51	Isoleucine	0.91 (t),1.00 (d), 1.25 (m), 1.47 (m), 1.97 (m), 3.65 (d)	0.55 ± 0.31	0.65 ± 0.13	0.33 ± 0.19	0.39 ± 0.08

### 2.3. Statistical Analysis

The chemical identities (*i.e.*, metabolites) of the observed ^1^H-NMR peaks were assigned based on both literature reports [30,31,32], and NMR software Chenomx (NMR suite 7.1, Professional) that contains a database of about 310 common metabolites associated with mammals and bacteria. Metabolites assignments were summarized in Table 1 and Table 2.

**Table 2:** Peak Assignments of Metabolites in HR-MAS NMR.

	Peaks’ Signals	Chemical Shifts	Relative Conc. Mean ± SD	
		(ppm)	Tumor	Control
3	Methyl	0.88	0.87 ± 0.20	1.00 ± 0.06
7	Lipid CH2	1.28 (m)	1.49 ± 0.32	3.02 ± 0.71
13	Choline	3.22–3.24 (m)	0.68 ± 0.10	0.73 ± 0.05
17	Glycerol	4.16–4.30 (m)	0.02 ± 0.01	0.04 ± 0.02
18	PhosphatidylCholine	4.35 (m)	0.04 ± 0.01	0.08 ± 0.03
25	Lactate	1.32 (d), 4.2 (q)	0.16 ± 0.03	0.49 ± 0.28
26	Alanine	1.47 (d), 3.77 (m)	0.09 ± 0.01	0.06 ± 0.01
28	Glutamate	2.05 (m)	0.02 ± 0.01	0.01 ± 0.01
35	Glycine	3.55 (s)	0.32 ± 0.14	0.46 ± 0.21

*Estimation of absolute peak area in ^1^H HR-MAS NMR*: Spectral fitting of each HR-MAS NMR spectrum was carried out by deconvoluting the spectrum using mixed Gaussian and Lorentzian lineshape using commercially available software MestRenova (Version 6.0.4). Spectral fitting generated the absolute peak area of each peak relative to the spectrometer standard that was then used to calculate the estimate absolute peak area normalized to unit weight of tissue sample and unit scan. The good fit of solid HR-MAS is illustrated in the supplemental Figure S3, where the error of residual in fitting/deconvoluting with line widths of 5–30 Hz is only slightly over noise. For example, the highest error of residual long chain methylene (CH_2_)n group, located at about 1.28 ppm, is below 1%.

For ^1^H HR-MAS NMR spectrum, the strategy of the estimated absolute peak area normalized to per unit weight of tissue sample and per scan was used for comparison between the samples, which was obtained using the following strategy. The weight of the samples loaded into the MAS rotor for each sample was recorded during sample loading. The matching and tuning conditions of the RF circuit of the probe were set the same using a network analyzer. All other experimental conditions were kept identical from sample to sample. In this way, the absolute peak area of each spectral peak obtained by spectral fitting, to be discussed below, against the spectrometer standard is directly proportional to the sample weight and the number of accumulation numbers. The estimates of absolute peak area per unit weight of tissue sample scaled to per unit scan were then obtained by dividing the absolute peak area obtained from spectral fitting by the weight of liver tissues and the number of accumulation numbers. The results are listed in Table 2. The normalized estimated absolute metabolite peak area can then be quantitatively compared between samples.

*Estimation of absolute concentration in high resolution liquid state ^1^H-NMR*: From the Sample Preparation section, it is known that the extracted metabolites, either water soluble or lipid soluble, were dissolved in 600 µL of either D_2_O containing 0.5 mM TSP (sodium salt, 3-trimethylsilylpropionic acid) or CDCl_3_ containing 0.03% v/v TMS. Of the 600 µL, 550 µL was loaded into NMR tube for metabolic profiling work. The following two steps were employed to obtain the estimates of the absolute concentration. Firstly, each metabolite spectrum was fit using the well-established method provided by Chenomx (NMR suite 7.1, Professional). The peaks were fitted with line widths of 2–6 Hz based on good fits to the metabolite data. Well established protocols in Chenomx were followed to obtain the metabolite concentrations in the NMR sample tube of either the water or the lipid soluble liquid state ^1^H-NMR spectra based on the known internal TSP or TMS concentration. Secondly, the metabolite concentrations were further scaled to unit weight of wet liver tissue used for extraction so that the estimates of absolute metabolite concentration in each liquid state NMR spectrum could be directly compared between different samples. Using the commonly accepted extraction efficiency of 95%, the estimates of absolute metabolite concentration for a particular metabolite is calculated using the following equation:

Estimates of absolute Concentration in tissue = (1/0.95) * Concentrations in NMR tube _metabolite_* 600 uL/Vol. _tissue_ (1)

where tissue volume can be estimated using the density of H_2_O, *i.e.*, 1.0 g/mL. The estimates of the absolute concentrations for both the lipid and the water soluble metabolites obtained from liquid state NMR metabolic profiling experiments are summarized in Table 1.

*The estimates of relative concentrations*: For both ^1^H HR-MAS NMR on intact tissues and liquid state ^1^H-NMR on tissue extracts, the estimates of relative concentrations of metabolites were calculated as the integrated signal (peak area) of one region divided by total integrated signal from all the metabolites.

*Multivariate analysis,* i.e., *the Principal component analysis (PCA)*: PCA was carried out using software Unscrambler X (Version 10.1). In brief, PCA is a technique that is performed without sample class. And the principal components are plotted to illustrate degrees of variance between the principal components (PCs), also known as factors in a much simpler two dimensional format, allowing the analyst to observe any clustering or similarities that exist within a data set and thus recognize patterns. Following statistical analysis of melanoma and control using PCA, data for the first two principal components were displayed graphically as a set of scores (PC-1 *versus* PC-2) illustrating clustering among data points and complete separation of classes. Loadings cannot be interpreted without scores, and *vice versa*. Correlation loadings are computed for each variable for the displayed principal components (factors). In addition, the plot contains two ellipses to help check how much variance is taken into account. The outer ellipse is the unit-circle and indicates 100% explained variance. The inner ellipse indicates 50% of explained variance. The importance of individual variables is visualized more clearly in the correlation loadings plot compared to the standard loadings plot. Loadings describe the data structure in terms of variable contributions and correlations. Every variable analyzed has a loading on each PC, which reflects how much the individual variable contributes to that PC, and how well the PC takes into account the variation contained in a variable. Cross validation is applied as it also gives the ability to apply Martens’ uncertainty test (MUT) [33] for an independent test set for validation. With cross validation, the same samples are used both for model estimation and testing. “Mean centering”, was used for all the PCA analysis in this work.

## 3. Results

### 3.1. The Relative Intensities of Metabolites in NMR Spectroscopy

**Figure 1:**
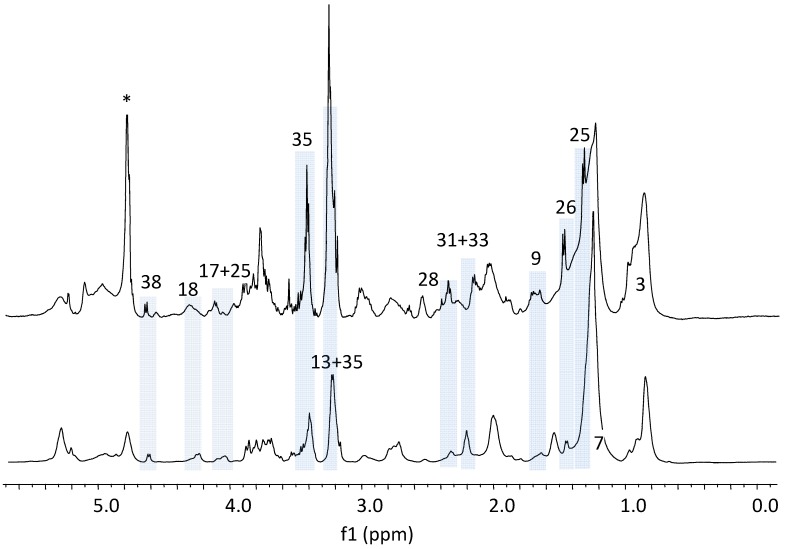
Representative ^1^H HR-MAS NMR Spectra of Intact Livers. Bottom trace: Control; Top trace: Tumor. Spectral assignments: 3.Lipids CH_3_; 7.Lipids (CH_2_)_n_; 9.Lipids CH_2_CH_2_CO; 13.Choline N(CH_3_)_3_; 25.Lactate; 26.Alanine; 28.Glutamate; 31.Glutathione; 33.Creatine + Creatinine; 35.Taurine; 38.β-Glucose. “*” denotes residual water signal. The doublets from lactate (d, J = 7.8 Hz) (peak “25”) and alanine (d, J = 7.7 Hz) (peak “26”) are the sharpest peaks in the spectra of melanoma liver, but their relative spectral intensities are much lower and their peak widths are much broader in the spectra of the control group when using lipid peak 7 at 1.28 ppm as the intensity reference. The relative intensities of peaks 13 (choline), 17 (glycerol ester), 18 (phosphatidylcholine), 25 (lactate), 26 (alanine), 28 (glutamate) and 35 (taurine) are much higher in the metastatic melanoma group than the ones in the control group with peak 7 (lipid CH_2_ group). Then grey rectangular areas in the Figure highlight those metabolites that are discussed in the text.

In the ^1^H HR-MAS NMR spectra on intact liver tissues, the peaks from metastatic melanoma group are visually different from those in the control group (Figure 1). The doublets from lactate (d, J = 7.8 Hz) (peak “25”) and alanine (d, J = 7.7 Hz) (peak “26”) are the obvious sharpest peaks in the spectra of melanoma liver, but their relative spectral intensities are much lower and their peak widths are much broader in the spectra of the control group when using lipid peak 7 at 1.28 ppm as the intensity reference. The relative intensities of peaks 13 (choline), 17 (glycerol ester), 18 (phosphatidylcholine, PtdCho), 25 (lactate), 26 (alanine), 28 (glutamate) and 35 (taurine) are much higher in the metastatic melanoma group than the ones in the control group with peak 7 (long chain lipid (**CH_2_**)_n_ group) as the reference. These results matched well with the literature reports on primary and secondary melanoma of lymph nodes using *in vivo* and *ex vivo* proton NMR spectroscopy [12].

**Figure 2:**
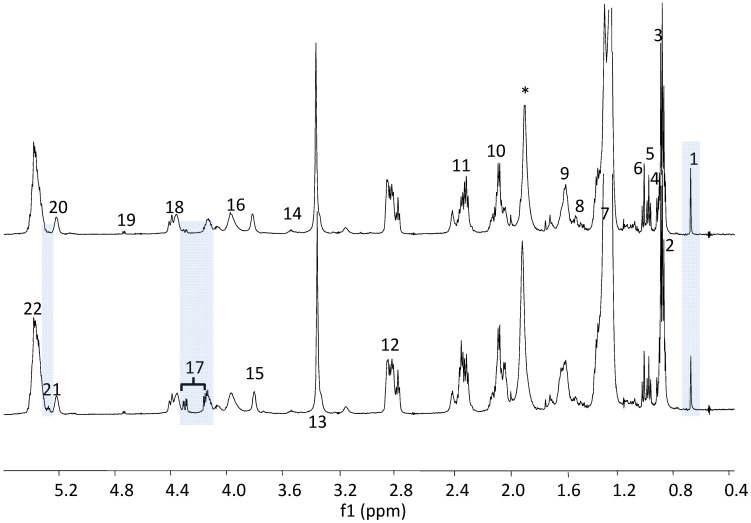
^1^H-NMR Spectra of Lipid Metabolites, Bottom: Control; Top: Tumor.

**Figure 3:**
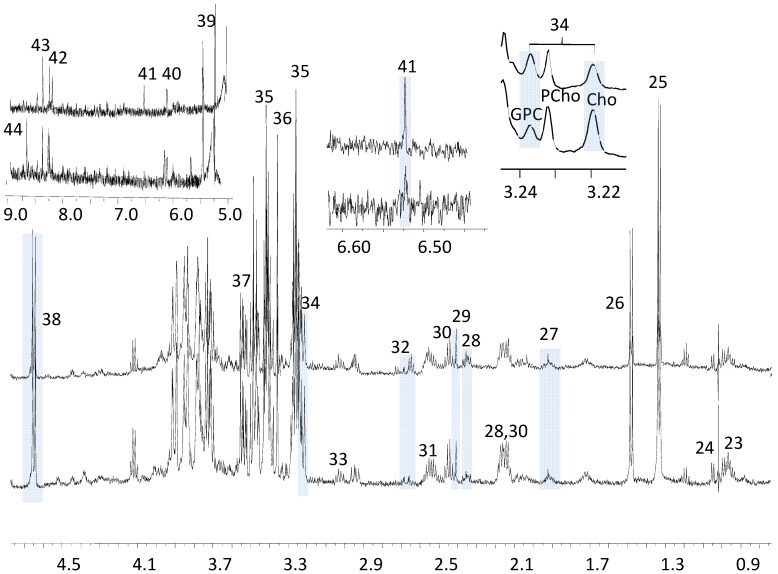
^1^H-NMR Spectra of Selected Hydrophilic Metabolites, Bottom: Control; Top: Tumor. (D_2_O, TSP). Peak assignments are: 23.Leucine; 24.Valine; 25.Lactate; 26.Alanine; 27.Acetate; 28.Glutamate; 29.Succinate; 30.Glutamine; 31.Glutathione; 32.Citrate; 33.Creatine + Creatinine; 34.Total choline; 35.Taurine; 36.Proline + Inositol; 37.Glycine; 38.β-Glucose; 39.α-Glucose; 40.Inosine derivatives; 41.Fumarate; 42.ATP/ADP; 43.Formate; 44.-NH. Metabolites like 29 (succinate), 32 (citrate), 41 (fumarate), 23 (leucine, isoleucine), 24 (valine), 26 (alanine), 37 (glycine) and other peaks like 25 (lactate), 34 (choline), 35 (taurine), 38 (glucose) were all observed in similar relative concentrations in both groups, but most noticeable changes were the ratio of glycerophosphocholine (GPC) to choline (Cho) and the concentration of fumarate between those two groups. The grey rectangular areas in the Figure highlight those metabolites that are discussed in the text.

Using the technique of HR-MAS NMR both the water soluble (hydrophilic) and the lipid soluble (hydrophobic) metabolites are observed in a single spectrum. To highlight their respective contributions, high resolution liquid state ^1^H-NMR spectra were acquired separately on both the water and the lipid soluble metabolites from tissue extracts. The lipid soluble metabolites extracted from liver tissues of the melanoma group and the control group both have similar ^1^H-NMR spectral features (Figure 2). The long chain methylene peaks 7, 10, 11, 12, methyl peaks 2, 3, 4 and choline peak 13 in the melanoma group are barely changed in relative concentrations compared to the control group, even though choline was previously thought to be one of most important biomarkers in cancer [34,35]. The only noticeable difference between the groups is seen in the glycerol ester (peaks 17 and 21) in the ^1^H-NMR; the melanoma group has relatively lower glycerol levels than the control. Another observation is that the cholesterol, methyl group peak 1 (cholesterol 18-CH_3_) in the spectra is quite intense (1.5–2 times) in the tumor as compared to the control, with peak 7 (lipids (CH_2_)n) used as an internal peak intensity reference. Cholesterol has previously been associated with transfection mediated by cholesterol-based cationic liposomes and regulation of melanogenesis in the tumor proliferations and metastases [36,37,38]. The ^1^H-NMR spectra of metabolites of the hydrophilic (water soluble) fraction were also compared, and all peaks were assigned and quantified using Chenomx software. We did not find a noticeable difference in relative peak intensities between the metastatic melanoma and the control groups (Figure 3); metabolites like 29 (succinate), 32 (citrate), 41 (fumarate), 23 (leucine, isoleucine), 24 (valine), 26 (alanine), 37 (glycine) and other peaks like 25 (lactate), 34 (choline), 35 (taurine), 38 (glucose) were all observed in similar concentrations in both groups (Figure 3). Therefore, similar to the findings from ^1^H HR-MAS using only relative concentrations of metabolites in the ^1^H-NMR spectra, an unambiguous set of metabolites associated with metastatic melanoma is difficult to determine, albeit some visual differences can be noticed.

1.Cholesterol 18-**CH_3_**; 2.Cholesterol 26, 27-**CH_3_**; 3.Lipids **CH_3_**; 4.Cholesterol 21-**CH_3_**; 5.Ω_3_ **CH_3_**; 6.Free cholesterol 19-**CH_3_**; 7.Lipids (**CH_2_**)n; 8.Cholesterol **CH_2_**; 9.Lipids **CH_2_**CH_2_CO; 10.Lipids **CH_2_**CH=C; 11.Lipids **CH_2_**CO; 12.Lipids =CH**CH_2_**CH=; 13.Choline N**(CH_3_)_3_**; 14.Cholesterol 3-**CH**OH; 15.Phosphatidylcholine N-**CH_2_**; 16.Glycerophospholipid backbone 3-**CH_2_**; 17. Glycerol backbone 1,3-**CH_2_**; 18.Phosphatidylcholine PO-**CH_2_**; 19.Esterified cholesterol 3-**CH**OH; 20. Glycerophospholipid backbone 2-**CH**; 21.Glycerol backbone 2-**CH**; 22.Lipid **CH=CH**. “*” denotes water residue. The long chain methylene peaks 7, 10, 11, 12, methyl peaks 2, 3, 4 and choline peak 13 in the melanoma group are barely changed in relative concentrations. The melanoma group has relatively lower glycerol levels than the control (peaks 17 and 21), but higher cholesterol level (peak 1). Then grey rectangular areas in the Figure highlight those metabolites that are discussed in the text.

### 3.2. The Estimates of Absolute Peak Intensities of Metabolites and Statistical Analysis

**Figure 4:**
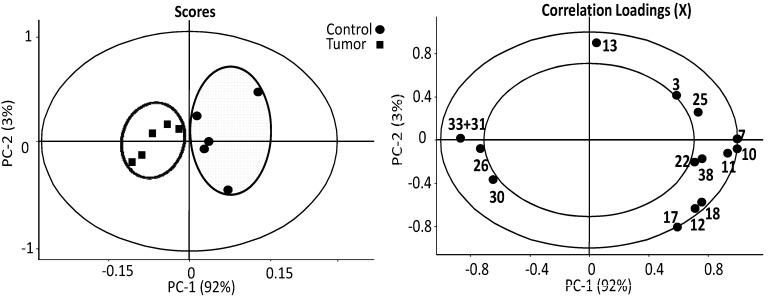
PCA analysis of the estimates of the absolute peak intensities from ^1^H HR-MAS NMR using all the metabolites/metabolite peaks that can be extracted from the spectra. The tumor and the control are clearly separated from each other in PC-1 axial. The decreased estimates of absolute intensities in tumor group are peaks 3 (methyl), 7, 10, 11, 12 (methylene), 13 (choline), 17 (glycerol backbone), 22 (double bonds), 25 (lactate) and 38 (β-glucose) with the correlation loadings in the positive PC-1. On the contrary, the negative PC-1 loading demonstrated that peaks 26 (alanine), 30 (glutamine) and 31 + 33 (creatine + glutathione) are elevated in the tumor group in estimates of absolute intensities. (number of samples in model) N = 10, (number of variables) K = 16, (number of latent components) A = 7, (model fit) R^2^ = 0.96, (predictive ability) Q^2^ = 0.95. The insider circle with radius r = 0.5, the outer one r = 1.

**Figure 5:**
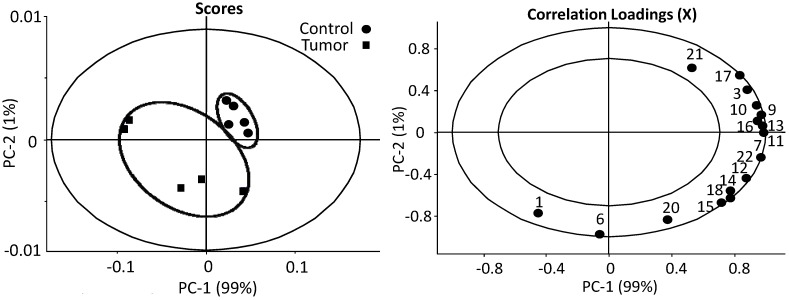
PCA analysis of the estimates of the absolute concentrations of lipid soluble metabolites from liquid state ^1^H-NMR, Different metabolites are labeled using the same numbers as in Figure 2. The only elevated lipid soluble metabolite in the tumor group is the cholesterol (peak 1), with negative correlation loadings in PC-1 axial. All other lipid metabolites, including glycerol backbone (peaks 17 and 21), linear lipid groups 7 (methylene), 9 (CH_2_CH_2_CO), 13 (Choline N(CH_3_)_3_), 15 (Phosphatidylcholine N-CH_2_), 18 (Phosphatidylcholine PO-CH_2_) and 22 (lipids CH=CH) are decreased according to the positive correlation loadings in PC-1. This result clearly indicates that the estimates of absolute concentrations of linear lipids including choline are decreased, while the cholesterol levels are increased in the metastatic melanoma group. (number of samples in model) N = 10, (number of variables) K = 17, (number of latent components) A = 7, (model fit) R^2^ = 0.98, (predictive ability) Q^2^ = 0.96. The insider circle with radius r = 0.5, the outer one r = 1.

The estimates of absolute peak intensities (^1^H HR-MAS NMR) were further analyzed using PCA and the results are summarized in Figure 4. The data from ^1^H HR-MAS NMR spectra show that the tumor and the control are clearly separated from each other in PC-1 axial. The decreased intensities in tumor group are peaks 3 (methyl), 7, 10, 11, 12 (methylene), 13 (choline), 17 (glycerol backbone), 22 (double bonds), 25 (lactate) and 38 (β-glucose) with the correlation loadings in the positive PC-1. On the contrary, the negative PC-1 loading demonstrated that peaks 26 (alanine), 30 (glutamine) and 31 + 33 (creatine + glutathione) are elevated in the tumor group in estimates of absolute intensities. These findings from ^1^H HR-MAS NMR spectra indicate significant changes or alternations in lipid oxidation and the energy metabolism associated with TCA cycle in melanoma metastasis liver. To further clarify the findings from HR-MAS NMR, complementary data from high resolution liquid ^1^H-NMR were further analyzed, taking advantage of the enhanced spectral resolution associated with liquid state ^1^H-NMR on liver extracts, and the results are summarized in Figure 5.

PCA analysis of the estimated absolute concentrations of lipid soluble metabolites showed that the tumor and the control are clustered into two major groups (Figure 5). The healthy group has highly clustered lipid contents, while the tumor group’s lipids vary. In scoring of PC-1 the tumor group has a negative score and the control has a positive score. The only clearly elevated lipid metabolite in the tumor group is the cholesterol characterized by peaks 1 (*i.e.*, cholesterol 18-CH_3_ that is unique to cholesterol), with negative correlation loadings in PC-1 axial (Figure 5) that has also been identified with elevated relative concentration in the discussion of Figure 2 earlier. It is also known from Table 1 that the concentrations determined by peaks 2, 4, 6 and 8 from cholesterol give consistent results. For example, peak 6 (free cholesterol) is found to have a strong negative loading (−0.8) in PC2 as well as a negative loading in PC-1, indicating elevation in the tumor samples. Since peak 6 and peak 1 both originate from cholesterol, it is not surprising, but a physical necessity, that these two peaks of the same metabolite behave similarly in the loadings plot. All other lipid metabolites, including glycerol backbone (peaks 17 and 21) that appear to be elevated in Figure 2, are in fact decreased according to the positive correlation loadings in PC-1; these consist of the linear lipid groups 7 (methylene), 9 (**CH_2_**CH_2_CO), 13 (Choline N**(CH_3_)_3_)**, 15 (Phosphatidylcholine N-**CH_2_)**, 18 (Phosphatidylcholine PO-**CH_2_**) and 22 (lipids CH=CH). This result clearly indicates that the estimates of absolute concentrations of linear lipids including choline are decreased, while the cholesterol levels are increased in the metastatic melanoma group.

**Figure 6:**
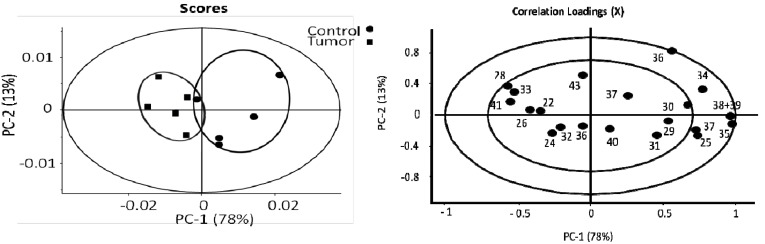
PCA analysis of the estimates of the absolute concentrations of the water soluble metabolites (hydrophilic metabolites) from liquid state ^1^H-NMR. Metabolites are labeled using the same numbers as in Figure 3. The positive correlation loading in PC-1 indicates decreased concentrations of 29 (succinate) and 38 + 39 (α, β-glucose); and negative PC-1 correlation loadings: 26 (alanine), 28 (glutamate), 33 (creatine), and 41 (fumarate) with increased concentrations. Melanoma and control could be separated in two groups in the score diagraph. (number of samples in model) N = 10, (number of variables) K = 20, (number of latent components) A = 7, (model fit) R^2^ = 0.91, (predictive ability) Q^2^ = 0.89. The insider circle with radius r = 0.5, the outer one r = 1.

Using the estimates of absolute concentrations of the water soluble metabolites, the tumor and the control groups are also separated with each other in the PC-1 score (Figure 6). We identified the following significant metabolites in the melanoma group, with negative PC-1 correlation loadings: 26 (alanine), 28 (glutamate), 33 (creatine), and 41 (fumarate). The positive correlation loading in PC-1 indicates decreased concentrations of 29 (succinate) and 38 + 39 (α, β-glucose). Those metabolites, such as glucose, fumarate, succinate, participate either directly or indirectly in the TCA cycle, which provides energy sources for cellular activities. With the preliminary analysis above, we then focused on lipid oxidation and the TCA pathway in order to pursue identification of interesting biomarkers for secondary melanoma [27,28,29].

The classical/traditional relative concentrations of the metabolites, defined as the relative peak area of a metabolite normalized to the total peak intensity of each spectrum, were also analyzed by PCA for comparing with the methods of the estimates of absolute concentration (see supplementary materials Figure S4 to S6). It is found that (i) the melanoma group can be distinguished from the controls using both the estimated absolute and the classical relative concentration approaches; (ii) For most of the metabolites, the increase or decrease in concentration are also consistent; and (iii) However, there are cases where estimates of absolute concentration gave much more accurate results. For example, the total choline (PC, GPC and free choline) in HR-MAS (Figure S4) is statistically and significantly increased with the classical relative concentration approach that is consistent with prior literature reports where relative concentrations were employed. However, in the estimated absolute concentration approach, the total choline is essentially not changed. This new finding, not obtainable with the relative concentration approach, clearly indicates the importance of employing the estimated absolute concentration for statistical analysis and the subsequent analysis for identifying possible biological pathways that are affected by a disease.

## 4. Discussion

We found that, in the ^1^H HR-MAS NMR spectra of melanoma, the relative intensities of metabolites such as peaks 13 (choline), 17 (glycerol ester), 25 (lactate), 26 (alanine), 28 (glutamate) and 35 (taurine) are much higher than those in the control, a result that is consistent with previous studies of lymph nodes of primary and secondary melanoma [12]. Furthermore, the relative peak intensity ratio of glycerophosphocholine (GPC) (3.3 ppm) and phosphocholine (PC) (3.2 ppm) is increased by about 1.5 fold in the melanoma group as compared to controls. The relative ratio of GPC to PC has been suggested as a bio-indicator of several types of cancers [39,40,41]. Up until now, a significant part of the work in the field of NMR metabolomics still uses relative peak intensities/relative metabolite concentrations. Therefore, based on prior suggestions [12,42], ^1^H HR-MAS NMR analysis of intact liver tissue using relative spectral intensities along (namely, the traditional ^1^H HR-MAS) could be used in diagnosing secondary melanoma tumor clinically. But the traditional HR-MAS does not give the estimates of absolute intensities of the metabolites since the long chain lipid (-(CH_2_)_n_-) signal, *i.e.*, peak 7, located at about 1.28 ppm is frequently used as the intensity reference. This is only meaningful when the 1.28 ppm peak is unchanged.

We found that the 1.28 ppm peak was actually down regulated during the development of melanoma metastasis. In Bourne *et al*.’s report the relative elevations in the aforementioned metabolites were thought to be relevant to melanoma pathology; however, it was not explained why melanoma tissue has relatively higher concentrations of these metabolites than normal tissues. Possible reasons for the relative elevations in these metabolites could be: (1) a decrease in the absolute concentration of the lipid peak 7 at 1.28 ppm gives the false impression of increase in other metabolites when this peak is inappropriately used as intensity reference; or (2) these metabolites had higher estimates of absolute concentration; or (3) these metabolites were not elevated but the estimates of absolute concentrations of NMR observable lipids in intact tissues were decreased; or (4) these metabolites concentrations including lipids were all increased, but lipids increased less relative to other metabolites in the metastatic tumor.

After quantifying the estimates of absolute intensities of the metabolites by wet tissue mass and analyzing both solid ^1^H HR-MAS NMR and liquid ^1^H-NMR data using PCA, we found that the estimates of absolute concentration of linear lipids is actually much lower in the liver of secondary melanoma than that in the liver of the control. We also found that the melanoma tumor group has lower estimates of absolute concentration of glycerol derivatives than the control group. These results indicate increased consumption of energy for tumor growth and accompanying DNA/RNA syntheses associated with the lipid metabolism pathway. It is known that the lipid content of the cell mediates the expression of genes involved in cell migration, invasion and angiogenesis contributing to tumor metastasis by modulating phosphorylation and acetylation of proteins [43]. Furthermore, we found higher estimates of absolute cholesterol concentrations in the melanoma compared with controls. Increased cholesterol concentrations are associated with proliferating cells and metastases of melanoma as previously stated [36,44]. Since the transfer of long chain fatty acid into mitochondria is the rate determining step in β-oxidation [45,46,47], the enzymes responsible for transfer and β-oxidation of fatty acids (like CPT-1) could be the targets of antitumor drugs [48,49,50,51]. An increase in total choline is generally recognized as a well-known metabolic event in tumor progression [34,35,52].

Instead of quantitation using our estimated absolution peak intensities (HR-MAS) or the estimated absolution concentrations of metabolites (liquid state NMR), many studies use relative metabolite peak area ratios to describe changes in metabolic profile. An disadvantage of relative peak area ratios is that the effects of a general reduction in the measured metabolite concentrations due to variations in cellular density will not be observed, and lactate was not statistically significant different between grades due to its high variability within each tumor group [53]. Utilizing ^1^H HR-MAS NMR with relative peak area ratios, our results show that the relative content of total choline is statistically higher in the liver of secondary tumor with lipids at 1.28 ppm as the peak intensity reference, but we now know that the estimates of absolute intensity of total choline is actually lower in melanoma mice (Figure 4) when quantification using estimates of absolute metabolite concentration is employed. This contrast in results obtained for total choline, which gives higher relative intensities in ^1^H HR-MAS NMR and lower estimates of absolute intensities in both HR-MAS and liquid state ^1^H-NMR, explains previous results that total choline concentration was dependent on the quantification method used [42]. Interestingly the ratio of glycerophosphocholine (3.3 ppm) and phosphocholine (3.2 ppm) is different between melanoma and control group in the aqueous fraction (Figure 3); the melanoma group has higher ratios of glycerophosphocholine to phosphocholine (1:1) and glyerophosphocholine to choline (about 1:1.5), which indicates the possibility of an alteration in glycerophosphodiesterase (such as EDI3), which regulates interconversions between glycerophosphocholine and choline [52].

Furthermore, it has been found recently that glycerophosphodiesterase EDI3 cleaves glycerophosphocholine (GPC) to form choline and glycerol-3-phosphate (G3P), and choline is then further metabolized to phosphatidylcholine (PtdCho), the major lipid in membranes and a key player in membrane-mediated cell signaling [54]. Glycerol-3-phosphate, is a precursor molecule for several lipids with central roles in signaling, for example lysophosphatidic acid (LPA), phosphatidic acid (PA) and diacylglycerol (DAG), and LPA activates intracellular signaling pathways by binding to specific LPA receptors, including membrane-bound G protein-coupled receptors and the intracellular nuclear receptor, PPAR-γ [54]. The results suggest that EDI3 either directly generates signaling molecules or provides “membrane anchors” for downstream signaling factors, and so links choline metabolism to signaling activities resulting in a more malignant phenotype [54]. Enzyme EDI3 plays an important role in controlling GPC and choline metabolism, and the GPC/PC ratio can be corrected by inhibiting EDI3 activity resulting in a decreased migration capacity of tumor cells, indicating EDI3 as a possible target for therapeutic intervention [40]. The complex role of GPC remains poorly understood, but it has become increasingly clear that high, rather than low, GPC concentrations are associated with poor prognosis in breast cancer [55]. Furthermore, another study suggests that choline metabolism and tumor perfusion in brain metastases are interrelated due to the influence of the transcription factor HIF-1 [34]. These findings identified that EDI-3 participates in the regulation of choline phospholipid metabolism in ovarian, prostate, breast, brain and other metastatic cancers by modifying the ratio of GPC to PC, and interfering with lipid metabolism and cell signaling [54]. Further elucidation of GPC metabolism can lead to findings of new prognostic biomarkers or drug targets [55].

In analysis of the hydrophilic (water soluble) metabolites, we found lower estimates of absolute concentration of glucose in the melanoma group attributable to the elevated energy requirements for tumor growth (Figure 5). The estimate of absolute concentration of lactate observed in liquid ^1^H-NMR is also statistically lower in the melanoma group than in the controls. A higher estimate of absolute creatine concentration is observed in NMR spectra in the tumor group, reflecting the energy metabolism of tumor growth [56]. Taurine, observed in higher estimates of absolute concentration of melanoma group, is cytoprotective and affects the levels of the antioxidant enzymes in B16F10 melanoma cells [57]. An increase in alanine, decrease in succinate and consumption of glycine are seen as signals of energy production and nucleotide *de novo* synthesis [58]. The melanoma group also exhibits elevated glutamate concentrations. Glutamate can be converted into succinate through γ-Aminobutyric acid (GABA) pathway. Elevations in glutamate levels are observed in B16 melanoma, where glutamate functions as a signal messenger in melanocytic neoplasia [59]. A drug named Riluzole targeting the release of glutamate is presently in clinical trials and shows promising results [60]. Succinate, an intermediate of TCA cycle , is found in lower concentration in the melanoma group than in controls, and fumarate is found in higher concentrations. The significance of this finding has not been fully elucidated, but the genes encoding succinate dehydrogenase (SDH) and fumarate hydratase (FH) are thought to be major tumor suppressor genes [61,62]. Both TCA cycle enzymes encoded by these genes participate in the hypoxia-inducible factor (HIF) signaling pathway in tumor cell proliferation [50,62]. Reverse trends, with decreased fumarate and increased succinate concentrations, are found in AML cell line HL-60 when treated with combined drugs MPA + BEZ (medroxyprogesterone acetate and bezafibrate), suggesting a role for ROS (reactive oxygen species) in tumorigenesis [63]. 

**Figure 7:**
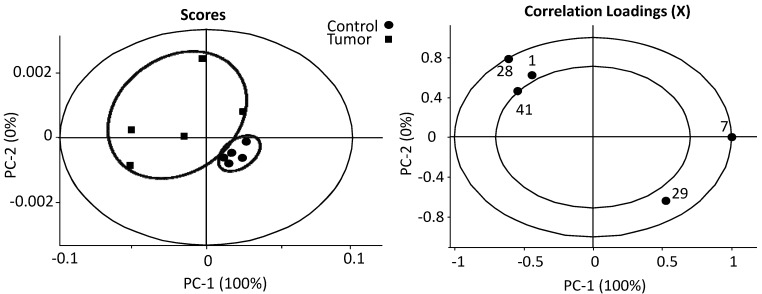
PCA analysis of important metabolites. Individual groups can be clustered into two groups from the first factor based on most changeable metabolites in melanoma, including both hydrophilic and hydrophobic metabolites. For secondary melanoma, the metabolites cholesterol (1), glutamate (28), fumarate (41) were increased with negative PC-1, and lipids (7) and succinate (28) and were decreased with positive PC-1.

Our findings from principal components analysis indicate that melanoma has effects on both lipid oxidation and the TCA cycle; from these findings, we have identified biomarkers which can separate the melanoma group from control groups (Figure 7), including linear lipids, cholesterol, glutamate, succinate and fumarate, where we can see that individuals can be clustered into two groups from the first factor. We found that, for secondary melanoma, the metabolites cholesterol, glutamate, and fumarate were increased, and lipids and succinate were decreased. Specifically, melanoma mice accumulate fumarate, and when the metabolite fumarate is increased, it inhibits PHDs (prolyl hydroxylases), leading to reduced hydroxylation of HIF-1α, which then stabilizes and activates the protein in thyroid carcinoma [64]. The activated HIF-1α results in the up regulation of target genes, which promote tumor cell survival and progression [14]. It is known that the deficiency of HIF promotes hypoxia-induced cell death in melanoma cells [65].

**Figure 8:**
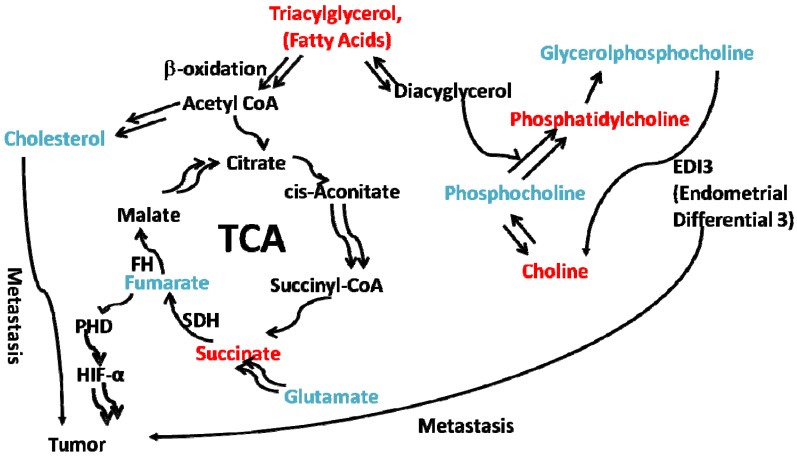
Proposed pathway in melanoma development, *i.e.*, the reactions between different metabolites. Here, all metabolites increased in the tumor group (as compared to the controls) are in blue, and all metabolites decreased in the tumor group (as compared to the controls) are in red, unchanged (including not detected) metabolites in the pathway, are in black.

Here, given the results of our metabolomics study of metastatic melanoma in the context of a review of current literature, we suggest that the accumulation of fumarate plays an important role both in TCA cycle and HIF signal pathway shown in Figure 8, where it further inhibits the PHDs and hence activates the HIF-1α to promote carcinoma cell proliferation, angiogenesis and metastases. The accumulation of fumarate is thought to be promoted by T_3_ (triiodothyronine), which inhibits fumarate dehydrogenase [64]. This possible link between TCA circle and the HIF-α signaling pathway suggested by our preliminary results in metabolomics of metastatic melanoma requires further study. Meanwhile, the interconversion of GPC/PC, lipid oxidation and γ-Aminobutyric acid (GABA) pathway, where glutamate can be converted into succinate in the TCA cycle, were also identified as a possible metabolic change in metastatic melanoma from our experiments, as shown in Figure 8.

## 5. Conclusions

We show that the combined use of ^1^H HR-MAS NMR metabolomics on intact tissues, liquid state NMR metabolomics on tissue extracts, and multivariate statistics analysis (*i.e.*, PCA) is a powerful systems biology tool for the study of secondary metastatic melanoma in mouse liver. In HR-MAS NMR, all metabolites are observed, regardless whether they are water soluble or lipid soluble. Liquid state NMR metabolomics, however, provides better spectral resolution than HR-MAS NMR. The results obtained from liquid state NMR on tissue extracts are consistent with those from HR-MAS NMR when quantification using estimates of absolute concentration of metabolite is employed. We have found that the melanoma group can be differentiated from its control group by PCA analysis of the estimates of absolute peak intensities or the estimates of absolute concentrations of metabolites from either ^1^H HR-MAS NMR on intact liver tissues or the liquid state ^1^H-NMR spectra on liver tissue extracts. In particular, we found that the estimates of absolute concentrations of alanine, glutamate, creatine, creatinine, fumarate and cholesterol are elevated whereas the estimates of absolute concentrations of succinate, glycine, glucose, and the family of linear lipids are decreased in the melanoma group when compared with controls. By family of linear lipids, we are referring to fatty acids (triacylglycerol, diacylglycerol and monoacylglycerol), as well as phosphatidylcholine. Furthermore, we found that the ratio of glycerophosphocholine to phosphocholine is increased by about 1.5 fold in the melanoma group compared with the controls, while the estimate of absolute concentration of total choline was actually lower in melanoma mice. The decrease in linear lipids indicates that the lipid biosynthesis pathway is altered as a result of secondary melanoma metastasis in liver. Since succinate, fumarate, and glutamate are key metabolites related to the TCA cycle, the decrease in succinate, and increase in fumarate and glutamate suggest the TCA cycle is affected by melanoma metastasis. These results, when combined with the finding of an increase in cholesterol levels, suggest the following picture in melanoma metastasis into liver. Linear lipid levels decrease due to an elevation in beta oxidation, producing acetyl CoA, which contributes both to an increase in the synthesis of cholesterol, and provides an energy source input for TCA cycle. The levels of succinate and fumarate in TCA cycle change and activate the related enzymes (SDH and FH); Fumarate is also implicated in control of the PHDs and HIF signal pathway, believed to be an important pathway in tumor development. These findings suggest a link between lipid oxidation, the TCA circle and the hypoxia-inducible factors (HIF) signal pathway in tumor metastases. This study indicates that the metabolic profile from NMR can be exploited as signature of malignancy and cell hypoxia. Enzymes participating in tumor metabolism pathway may be interesting drug targets in future chemotherapy; possible drug targets include mitochondrial succinate dehydrogenase (SDH), fumarate hydratase (FH), carnitine palmitoyltransferase (CPT), glycerophosphodiesterase (EDI3) and hypoxia-inducible factors (HIF).

## Supplementary Files

Supplementary File 1 Supplementary Materials (PDF, 898 KB)
