# Supplementary material for: Studies of Secondary Melanoma on C57BL/6J Mouse Liver Using 1H NMR Metabolomics
Source: Metabolites. 2013 Oct 31;3(4):1011–35. doi: 10.3390/metabo3041011 (PMC3937829; doi:10.3390/metabo3041011)

Supplementary Materials

Figure S1. Light microscopy images of livers: 20 × (Left: Tumor; Right: Control).

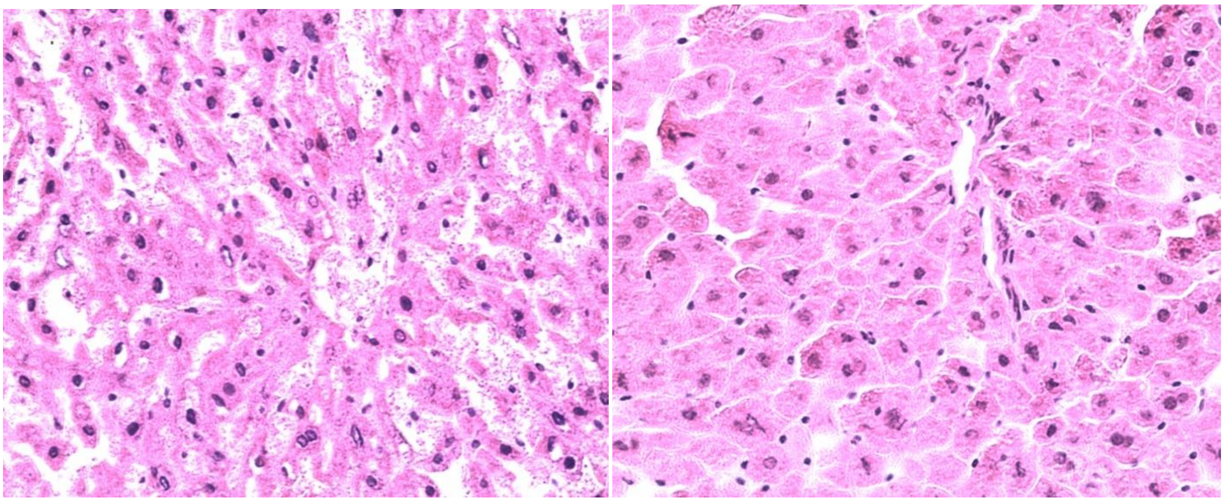

Melanocarcinoma masses were pigmented light to dark brown with ulceration and bleeding in some cases. Under microscope, there was variation in the cellular pattern composed of cells and alveoli. The cells were more or less uniformed in size and shape. The nuclei were vacuolated and contain large acidophilic nucleoli resembling including bodies. Melanin pigment was present and tended to be a uniform fine granularity, whereas in the chromatophores the granules were more irregular in size and shape. The cells of pegs loosened and incorporated in the dermal neoplasm. **The melanocarcinoma cells invaded to the epidermis.** The liver was soft with large dark red or purple colors, and oozed blood on cut. The central zones of lobules were congested and red, the color of its periphery was light brown. Microscopically there were dilatations and congestions of the central vein and adjacent central parts of the sinusoids. The hepatic cells in the central zone appeared atrophic or fatty degeneration. Focal necroses were occurring. There was no condensation or relative increase of connective tissue. Some area around the hepatic cells was occupied by a fine web of pink staining granular debris. **The melanocarcinoma cells invaded to the liver the tissue.** The findings from the imaging experiments are similar to previously reported [24,25].

Figure S2. Solid HR-MAS ^{1}HNMR of an intact control liver tissue. No significant metabolic changes were observed within 1h of data acquisition.

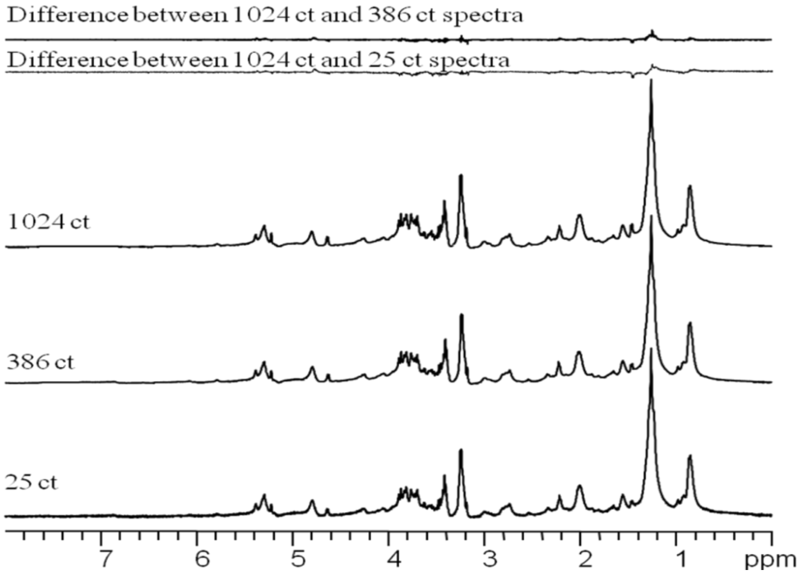

Figure S3. An Example of Deconvoluted Solid HR-MAS NMR spectrum acquired on a melanoma liver (blue line: fitting peak: magenta line: sum; red: residual). Error of residual (< 1%) in fitting/deconvoluting spectra is slightly over noise.

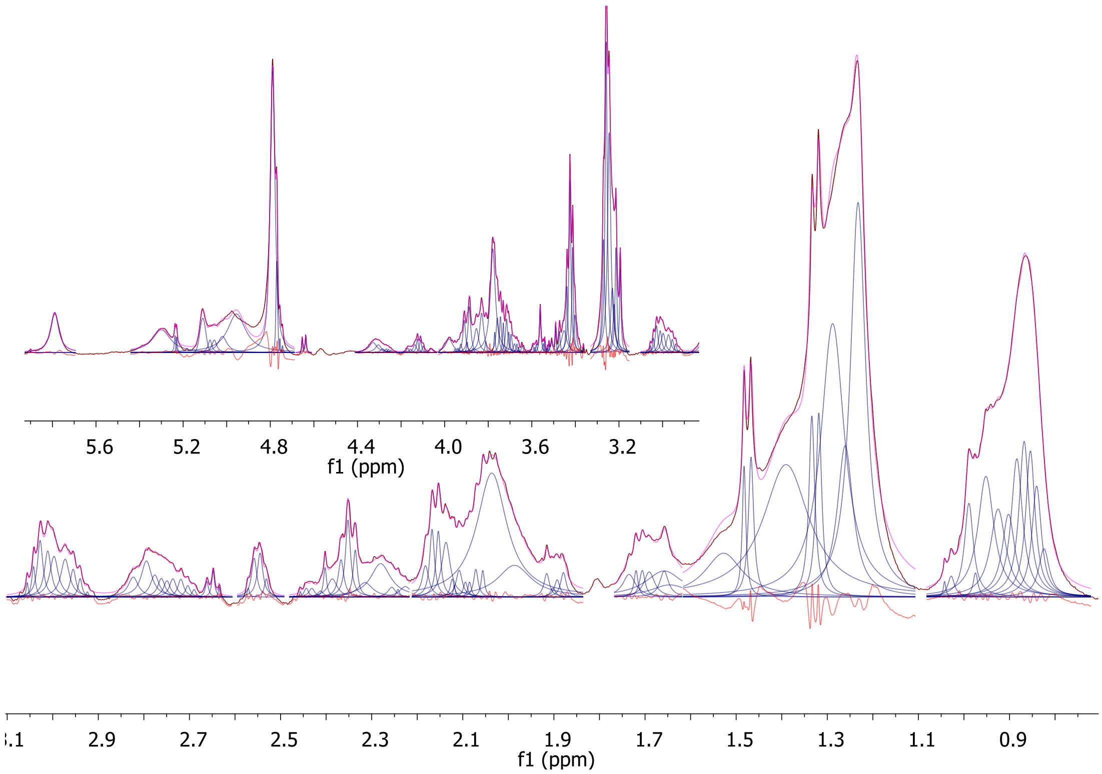

Figure S4. PCA from ^{1}H HR-MAS NMR Using Relative Concentration. (number of samples in model) N=10, (number of variables) K=17, (number of latent components) A=7, (model fit) R2=0.81, (predictive ability) Q2=0.69. The insider circle with radius r=0.5, the outer one r=1.

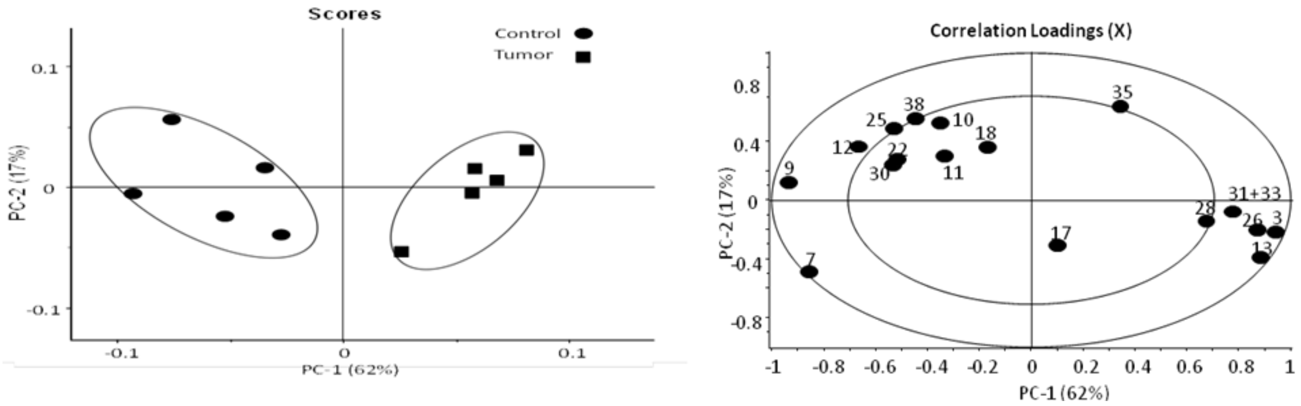

Figure S5. PCA from ^{1}H NMR Using Relative Concentration of Lipids Extracts. (number of samples in model) N = 10, (number of variables) K = 17, (number of latent components) A = 7, (model fit) R2 = 0.89, (predictive ability) Q2 = 0.79. The insider circle with radius r=0.5, the outer one r = 1.

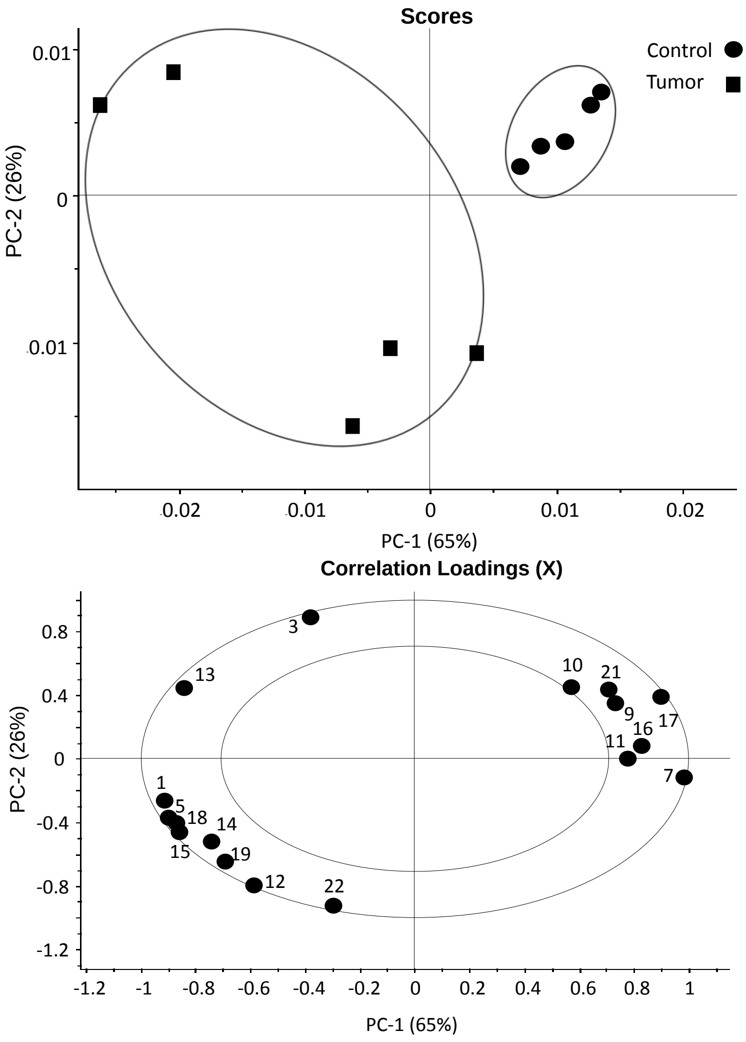

Figure S6. PCA from ^{1}H NMR Using Relative Concentration of Water Extracts. (numbers of samples in model) N = 10, (number of variables) K = 21, (number of latent components) A = 7, (model fit) R2 = 0.95, (predictive ability) Q2 = 0.90. The insider circle with radius r = 0.5, the outer one r = 1.

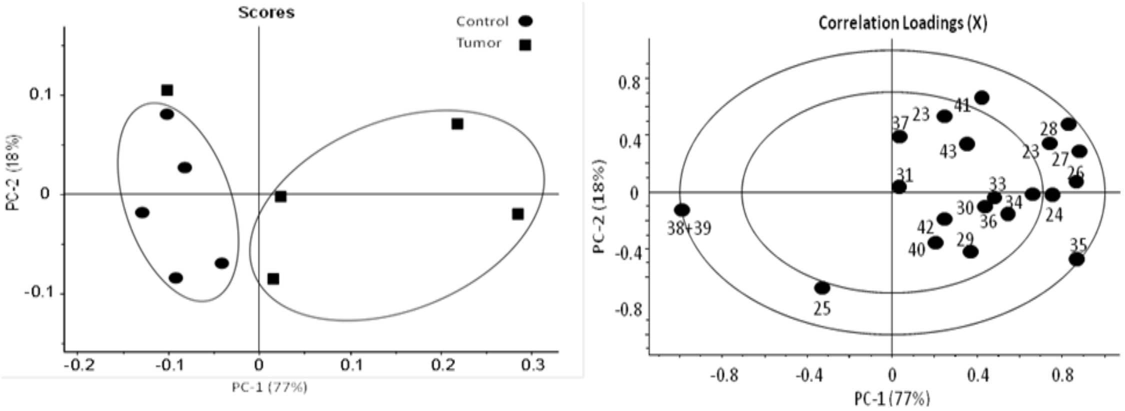

Supplement: Supplementary File 1 — Supplementary Materials (PDF, 898 KB) [file metabolites-03-01011-s001.pdf]
